# Supplementary material for: Cryo-EM structures of the human Elongator complex at work
Source: Nat Commun. 2024 May 15;15:4094. doi: 10.1038/s41467-024-48251-y (PMC11096365; doi:10.1038/s41467-024-48251-y)
Supplement: Supplementary file 1 — Supplementary Information [file 41467_2024_48251_MOESM1_ESM.pdf]

Supplementary Information

Cryo-EM structures of the human Elongator complex at work

Nour-el-Hana Abbassi[1,2,†], Marcin Jaciuk[1,†], David Scherf[3], Pauline Böhnert[3], Alexander Rau[4], Alexander Hammermeister[1], Michal Rawski[1,5], Paulina Indyka[1,5], Grzegorz Wazny[5,6], Andrzej Chramiec-Głąbik[1], Dominika Dobosz[1], Bozena Skupien-Rabian[1], Urszula Jankowska[1], Juri Rappsilber[4,7], Raffael Schaffrath[3,*] Ting-Yu Lin[1,††,*] and Sebastian Glatt[1,*]

Affiliations

[1] Małopolska Centre of Biotechnology (MCB), Jagiellonian University, Krakow, Poland

[2] Postgraduate School of Molecular Medicine, Medical University of Warsaw, Poland

[3] Institute for Biology, Department for Microbiology, University of Kassel, Kassel, Germany

[4] Bioanalytics, Institute of Biotechnology, Technical University of Berlin, Berlin, Germany

[5] SOLARIS National Synchrotron Radiation Centre, Jagiellonian University, Krakow, Poland

[6] Doctoral School of Exact and Natural Sciences, Jagiellonian University, Krakow, Poland

[7] Wellcome Centre for Cell Biology, University of Edinburgh, Edinburgh, UK

[*] correspondence to schaffrath@uni-kassel.de (R.S.) ting-yu.lin@durham.ac.uk (T-Y.L) and sebastian.glatt@uj.edu.pl (S.G.)

† These authors contributed equally to this work

†† present address: Department of Biosciences, Durham University, Durham, UK

Running title – Structure and function of human Elongator

Supplementary Method

Mass spectrometry analysis of acetylated peptides and peptide fragments

ELP123 complexes, including wild-type and mutants of ELP3, were produced and purified accordingly to the purification methods described in Methods. The purified ELP123 complexes were resolved in a 12% SDS-PAGE and protein bands were visualized using Coomassie blue staining. The ELP3 protein band was excised and protein identification was performed from gel bands. Samples (n=3, independent replicates) were prepared as described[1] with minor changes. Enzyme used for protein digestion was trypsin. Peptides were analyzed with LC/MS system consisting of nanoHPLC (UltiMate 3000 RSLCnano System, Thermo Fisher Scientific, Bremen, Germany) connected to Q Exactive mass spectrometer (Thermo Fisher Scientific) equipped with a Digital PicoView 550 nanospray source (New Objective). Peptides were applied onto a trap column (Acclaim PepMap 100 C18, 75 µm × 20 mm, 3 µm particle, 100 A° pore size, Thermo Fisher Scientific) in 2% acetonitrile with 0.05% TFA at a flow rate of 5 µl/min and further separated on analytical column (Acclaim PepMap RSLC C18, 75 µm × 500 mm, 2 µm particle, 100 A° pore size, Thermo Fisher Scientific) at 50°C with a 60-min gradient from 2 to 40% acetonitrile in 0.05% formic acid at a flow rate of 250 nl/min. The mass spectrometer was operated in a data-dependent mode using the Top8 method with 35 s of dynamic exclusion. MS and MS/MS data were acquired using Xcalibur (v. 3.1.66.10, Thermo Fisher Scientific). Full MS spectra (from m/z 300 to 1950) were acquired with a resolution of 70,000 at m/z 200 with an automatic gain control (AGC) target of 1e6. The MS/MS spectra were acquired with a resolution of 35,000 at m/z 200 with an AGC target of 3e6. The maximum ion accumulation times for the full MS and the MS/MS scans were 120 and 110 ms, respectively. For accurate mass measurements, the lock mass option was enabled. The collected LC-MS/MS data were processed with the Proteome Discoverer platform (v.1.4; Thermo Scientific) and searched using an in-house MASCOT server (v.2.5.1; Matrix Science, London, UK) against cRAP database (https://www.thegpm.org/crap/, released August 2019) supplemented with the sequences of ELP123 proteins as well as ELP3 protein mutants. The following parameters were applied in the database search: enzyme – trypsin; missed cleavages – up to 2; fixed modifications – carbamidomethyl (C); variable modifications – oxidation (M), acetylation (KSTY), phosphorylation (STY); peptide mass tolerance: 10 ppm; fragment mass tolerance: 20 mmu; minimum peptide length: 5 amino acids. Results were filtered to meet the Mascot significance threshold 0.01 on peptide level.

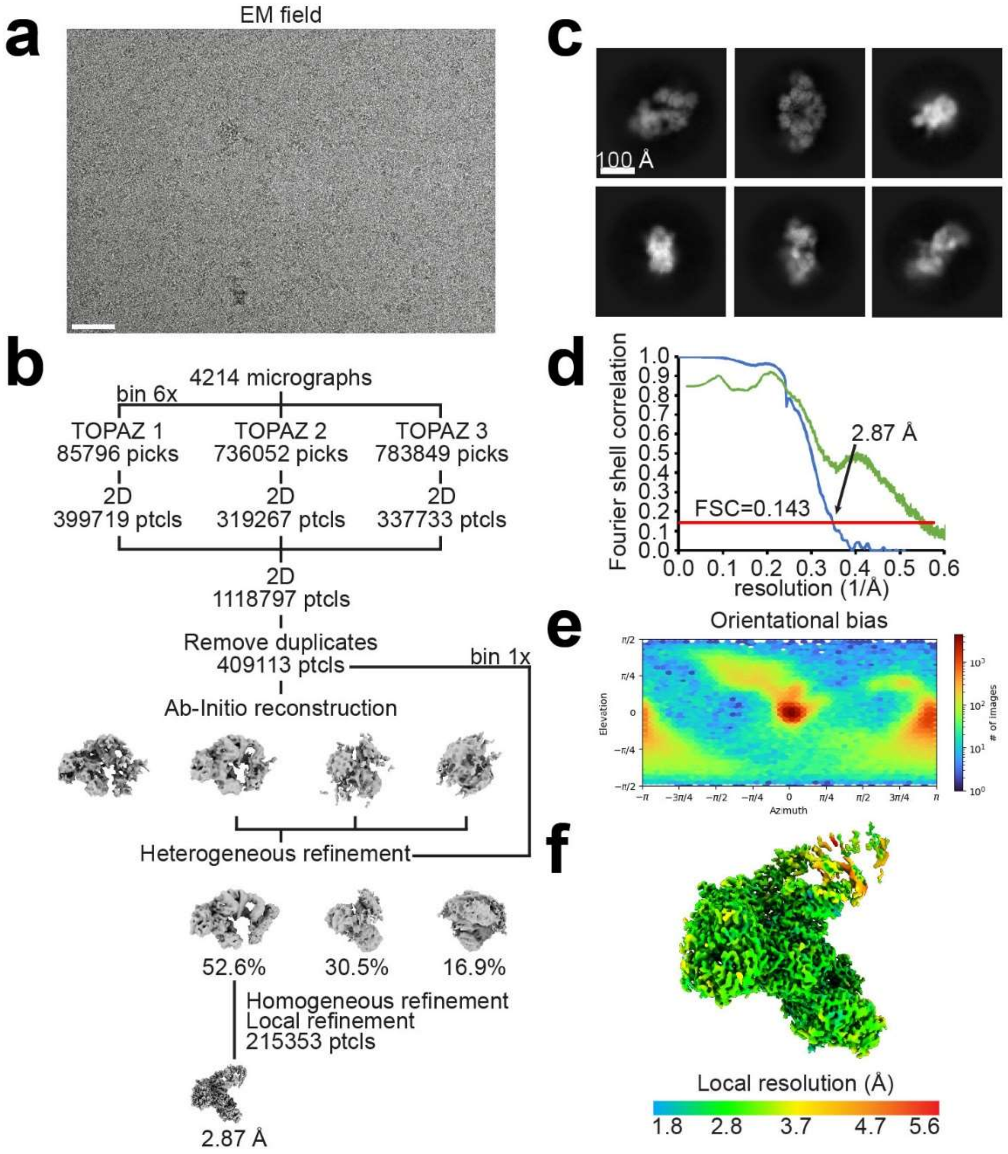

Fig. S1. ELP123–tRNA$^{Gln}_{UUG}$–acetyl-CoA cryo-EM reconstruction. (**a**) Representative cryo-EM field. Scale bar, 50 nm. (**b**) General processing pipeline. (**c**) Representative 2D classes. (**d**) Fourier Shell Correlation. FSC=0.143 where the blue line shows the refinement FSC and the green line shows the unmasked map-model FSC. (e) Orientational bias of the final density. (**f**) Local resolution estimation.

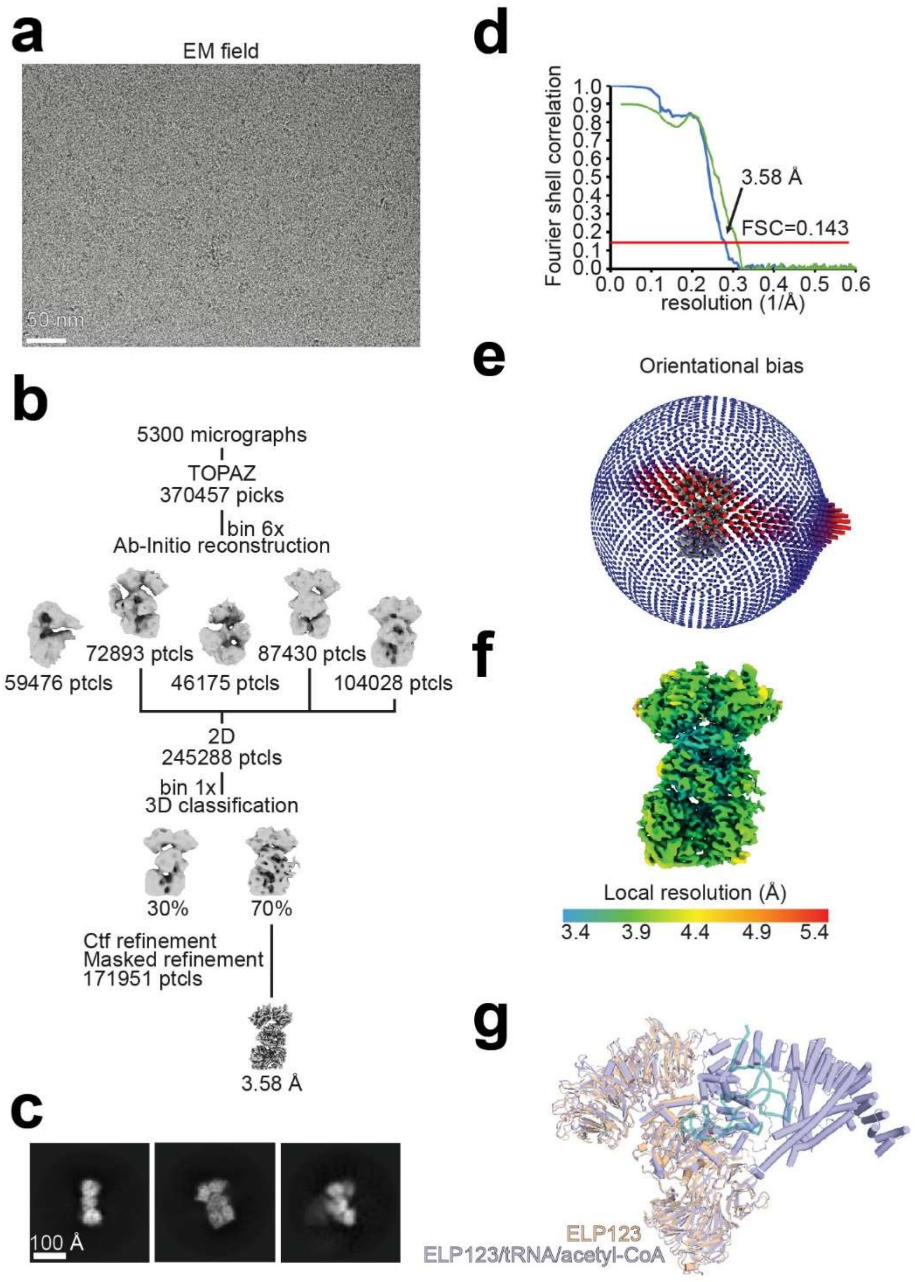

Fig. S2. ELP123 cryo-EM reconstruction. (**a**) Representative cryo-EM field. Scale bar, 50 nm. (**b**) General processing pipeline. (**c**) Representative 2D classes. (**d**) Fourier Shell Correlation. FSC=0.143 where the blue line shows the refinement FSC and the green line shows the unmasked map-model FSC. (**e**) Orientational bias of the final density. (**f**) Local resolution estimation. (**g**) Superimposition of the ELP123 and ELP123–tRNA$^{Gln}_{UUG}$–acetyl-CoA.

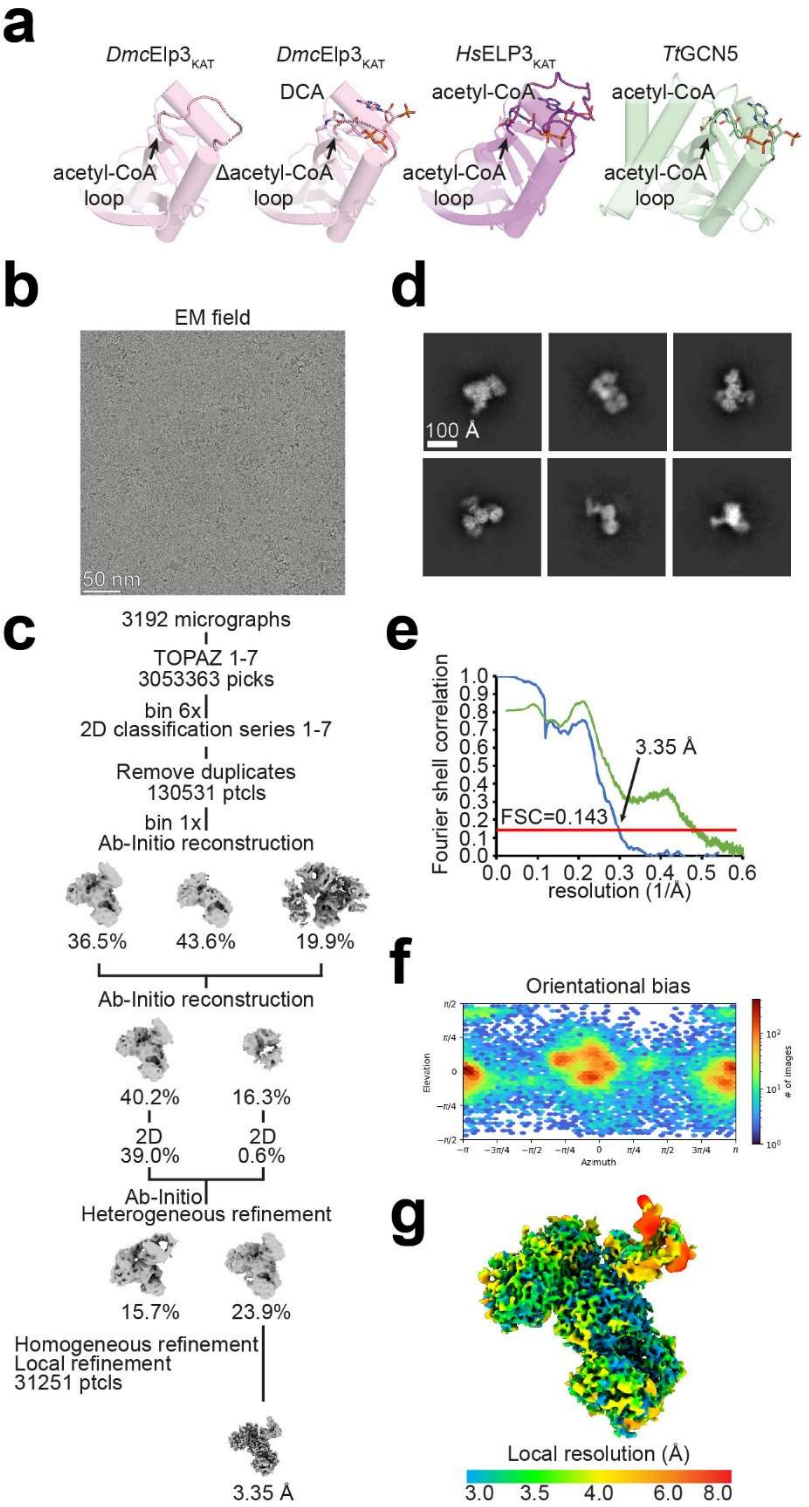

Fig. S3. ELP123-tRNA$^{Gln}_{UUG}$-ECA cryo-EM reconstruction. (**a**) KAT domain comparison between human ELP3 and *Dehalococcoides mccartyi* Elp3 (PDB ID 6IA6 and 5L7J) or *Tetrahymena thermophila* Gcn5 (PDB ID 1QSR). (**b**) Representative cryo-EM field. Scale bar, 50 nm. (**c**) General processing pipeline. (**d**) Representative 2D classes. (**e**) Fourier Shell Correlation. FSC=0.143 where the blue line shows the refinement FSC and the green line shows the unmasked map-model FSC. (**f**) Orientational bias of the final density. (**g**) Local resolution estimation.

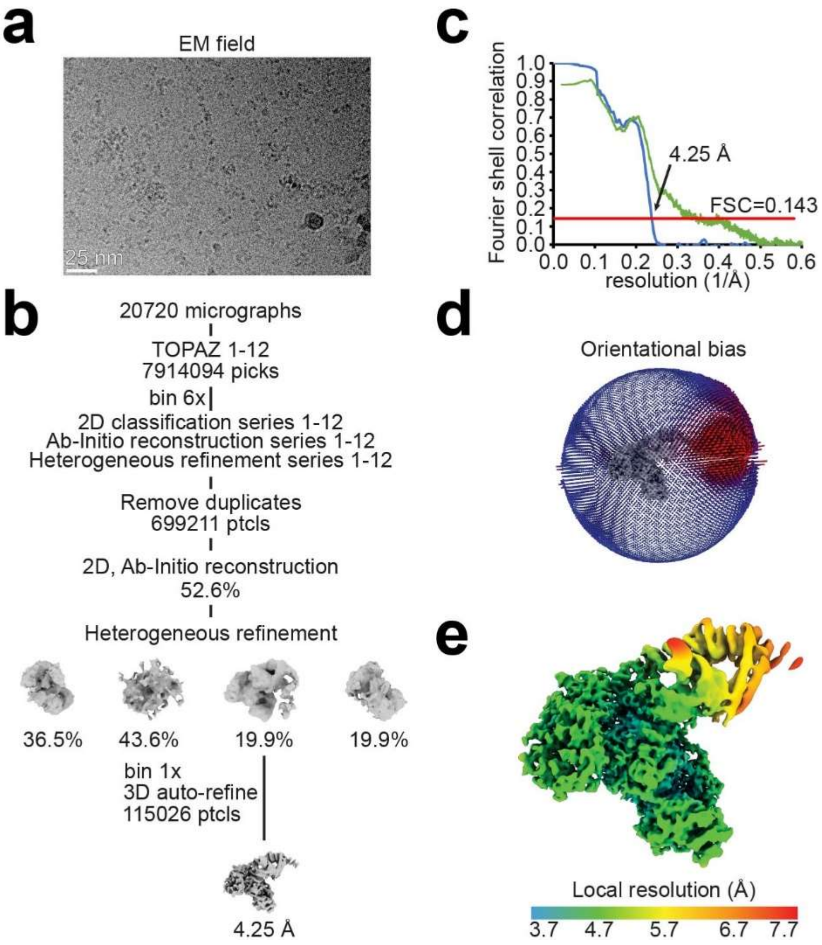

Fig. S4. ELP123–$tRNA^{Gln}_{UUG}$–DCA cryo-EM reconstruction. (**a**) Representative cryo-EM field. Scale bar, 50 nm. (**b**) General processing pipeline. (**c**) Fourier Shell Correlation. FSC=0.143 where the blue line shows the refinement FSC and the green line shows the unmasked map-model FSC. (**d**) Orientational bias of the final density. (**e**) Local resolution estimation.

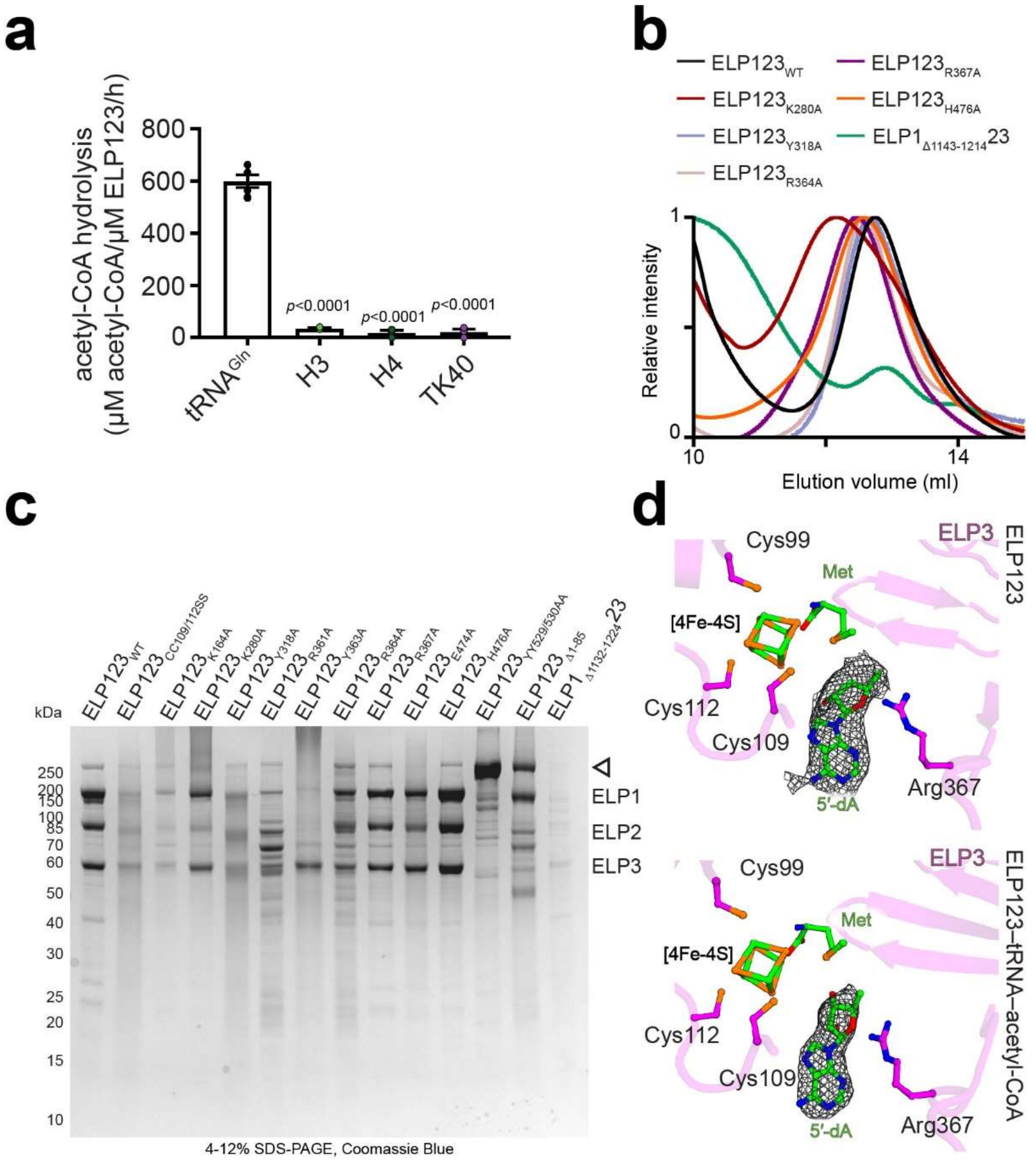

Fig S5. Purification of recombinant wild-type and various mutants of *Hs*ELP123. (**a**) Acetyl-CoA hydrolysis rates of ELP123 in the presence of $tRNA^{Gln}_{UUG}$ or protein peptides. n = 3 (independent experiments). Statistical analysis: one-way ANOVA. Statistically significant differences are indicated ($p \leq 0.0001$). Data are presented as mean values ± SEM. (**b**) SEC purification profiles of ELP123 WT and mutants. (**c**) SDS-PAGE gel analysis of ELP123 wild-type (WT) and mutant ELP123 subcomplex quality. The non-specifically co-purified Acc1/ACACA protein[2] is indicated by the triangle. (**d**) Close-up views of the iron-sulfur cluster, 5′-dA (respective densities are shown as mesh), and methionine in ELP123 and ELP123–tRNA–acetyl-CoA structures. The coordinating residues are highlighted. Source data are provided as a Source Data file.

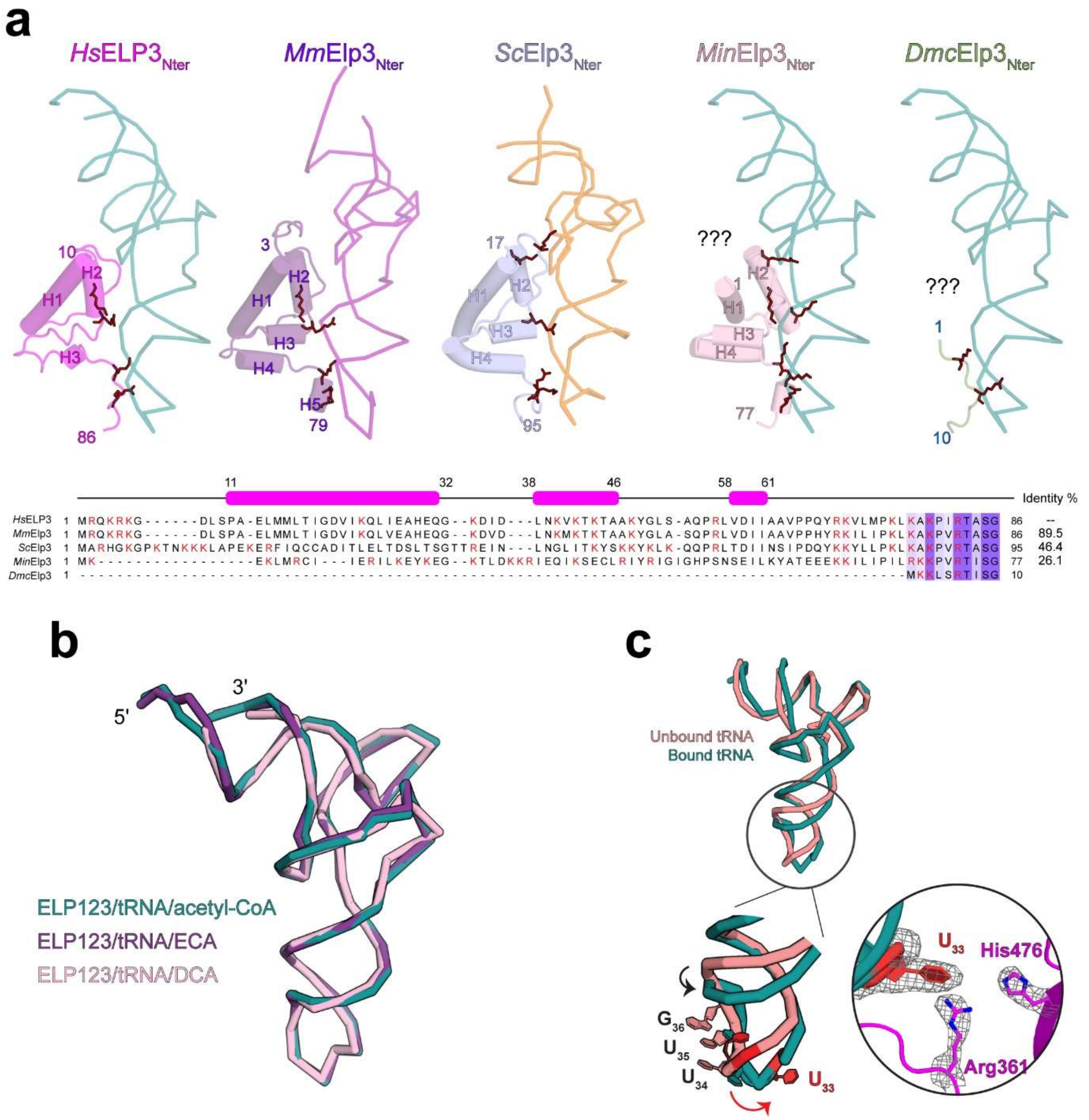

Fig S6. Comparisons of N-termini from various Elp3 sources and their interactions with tRNAs. (**a**) Structural comparison of N-termini of Elp3 homologs. Cartoon representation of each tRNA-bound structure: *Hs*ELP3 (this study, ELP123–tRNA–acetyl-CoA), *Sc*Elp3 (PDB 8ASW), *Mm*Elp3 (EMD-15625), *Min*Elp3 (AF2 prediction) and *Dmc*Elp3 (AF2 prediction). The basic residues in close contact with the tRNA are shown in sticks. Sequence alignment of Elp3 N-termini with the basic residues highlighted in red and identity to *Hs*ELP3 calculated with Blastp. The helices in the N-terminus of human ELP3 are shown and the amino acid numbering is indicated. (**b**) Superimpositions of the bound tRNA structures from ELP123–tRNA–acetyl-CoA, ELP123–tRNA–DCA and ELP123–tRNA–ECA. (**c**) tRNA unwinding upon binding to ELP123. Left: Superimpositions of unbound tRNA (PDB 1EHZ) and the bound tRNA (ELP123–tRNA–acetyl-CoA). Close-up view of the ASL. The U_{34}, U_{35} and G_{36} are shown while the movement of U_{33} is highlighted. Right: Close-up view of His476 and Arg361 residues facing U_{33}.

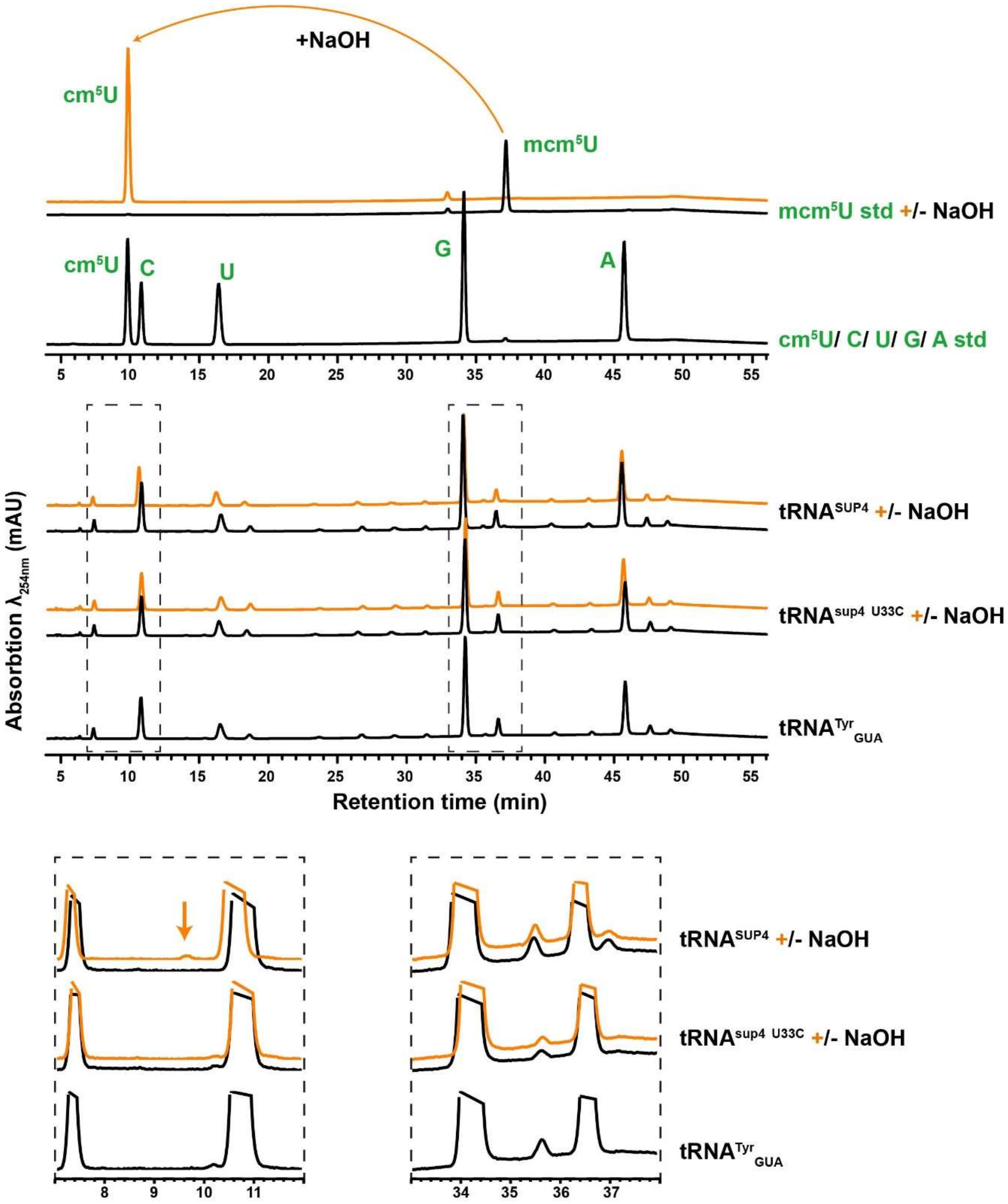

Fig S7. HPLC profiles of digested tRNA nucleosides. Analyses of mass standards (std; green) for U, C, G, A and modified uridine derivates (cm^5U and mcm^5U) are shown at the top. Hydroxylation treatment (+ NaOH, orange) converts mcm^5U to cm^5U. Individual $tRNA^{Tyr}_{GUA}$, $tRNA^{SUP4}$ and $tRNA^{sup4\ U33C}$ were isolated from various yeast strains overexpressing wild type and mutated *SUP4* tRNA. The purified tRNA was enzymatically hydrolyzed to nucleosides, dephosphorylated and subjected to HPLC analysis. At the bottom, close up views for the regions of interest are shown and the NaOH-dependent peak of cm^5U (converted mcm^5U) is highlighted by an orange arrow.

a

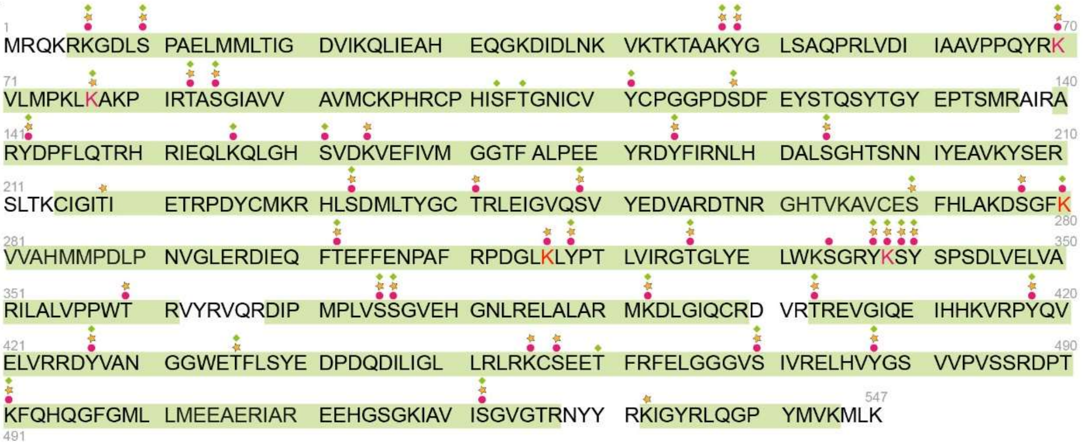

b

● ELP3 ☆ $ELP3_{K280A}$ ◆ $ELP3_{Y363A}$

	ELP3 Sum of PSMs	ELP3 Max ion score		$ELP3_{K280A}$ Sum of PSMs	$ELP3_{K280A}$ Max ion score		$ELP3_{Y363A}$ Sum of PSMs	$ELP3_{Y363A}$ Max ion score
K6/S10	369	118	K6/S10	358	108	K6/S10	329	108
K48/Y49	21	62	K48/Y49	22	52	K48/Y49	17	52
K70	5	27	K70	6	19	K70	3	19
K77	n.d.	--	K77	1	14	K77	1	15
T83/S85	56	100	T83/S85	50	84	T83/S85	40	87
S103/T105	n.d.	--	S103/T105	n.d.	--	S103/T105	6	39
Y111	30	74	Y111	n.d.	--	Y111	6	39
S118	n.d.	--	S118	4	42	S118	11	40
Y142	13	34	Y142	18	44	Y142	15	34
K156	1	17	K156	n.d.	--	K156	1	22
S161	1	31	S161	n.d.	--	S161	13	73
K164	5	43	K164	8	58	K164	n.d.	--
Y184	6	28	Y184	3	21	Y184	5	25
S194	4	29	S194	7	69	S194	8	58
T219	n.d.	--	T219	1	25	T219	n.d.	--
S233	5	38	S233	2	26	S233	12	59
T241	4	76	T241	4	50	T241	n.d.	--
S249	4	96	S249	6	26	S249	4	124
S270	n.d.	--	S270	4	38	S270	4	45
S277	4	49	S277	66	83	S277	n.d.	--
K280	2	49	K280	n.d.	--	K280	6	48
T302	8	43	T302	7	44	T302	6	37
K316	1	35	K316	1	38	K316	n.d.	--
Y318	13	24	Y318	26	24	Y318	20	26
T326	7	32	T326	6	41	T326	5	42
S334	2	61	S334	n.d.	--	S334	n.d.	--
Y337/K338	17	75	Y337/K338	26	59	Y337/K338	26	70
S339/Y340	244	77	S339/Y340	275	73	S339/Y340	233	82
T360	2	20	T360	4	49	T360	n.d.	--
S375/S376	5	55	S375/S376	13	65	S375/S376	34	62
K392	10	49	K392	6	37	K392	1	27
T403	11	57	T403	12	43	T403	14	52
Y418	10	35	Y418	9	34	Y418	7	35
Y427	20	134	Y427	22	121	Y427	32	99
T435	n.d.	--	T435	6	74	T435	18	66
K455	5	39	K455	5	52	K455	n.d.	--
S457	2	23	S457	3	41	S457	n.d.	--
T460	n.d.	--	T460	n.d.	--	T460	1	35
S470	1	48	S470	3	65	S470	3	70
Y478	1	22	Y478	20	54	Y478	15	56
K491	6	44	K491	2	53	K491	2	29
S522	3	45	S522	6	58	S522	6	54
K532	n.d.	--	K532	1	20	K532	n.d.	--

c

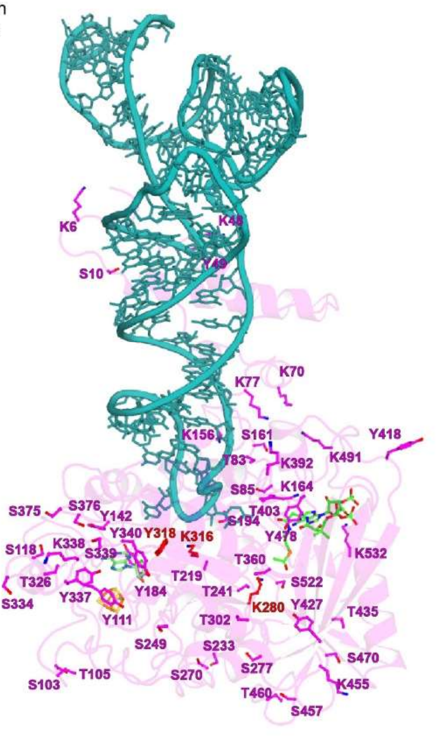

Fig S8. Mass spectrometry analysis of acetylation profiles of ELP3. (**a**) Scheme of *Hs*ELP3 protein sequence. The identified peptides by mass spectrometry are highlighted in green while the relevant acetylated lysine residues are shown in red. Above the modified residues are the colored symbols indicating in which condition they were found: ELP3, pink circle; $ELP3_{K280A}$, yellow star; and $ELP3_{Y363A}$, green diamond. (**b**) Tables of scores summarized from three independent mass spectrometry analyses. Ion score refers to the confidence level of the data, PSMs refers to the number of reads performed on each peptide. (**c**) Mapping the identified acetylated lysine residues in ELP3 and the bound tRNA. Lys280, Lys316 and Tyr318 are highlighted in red.

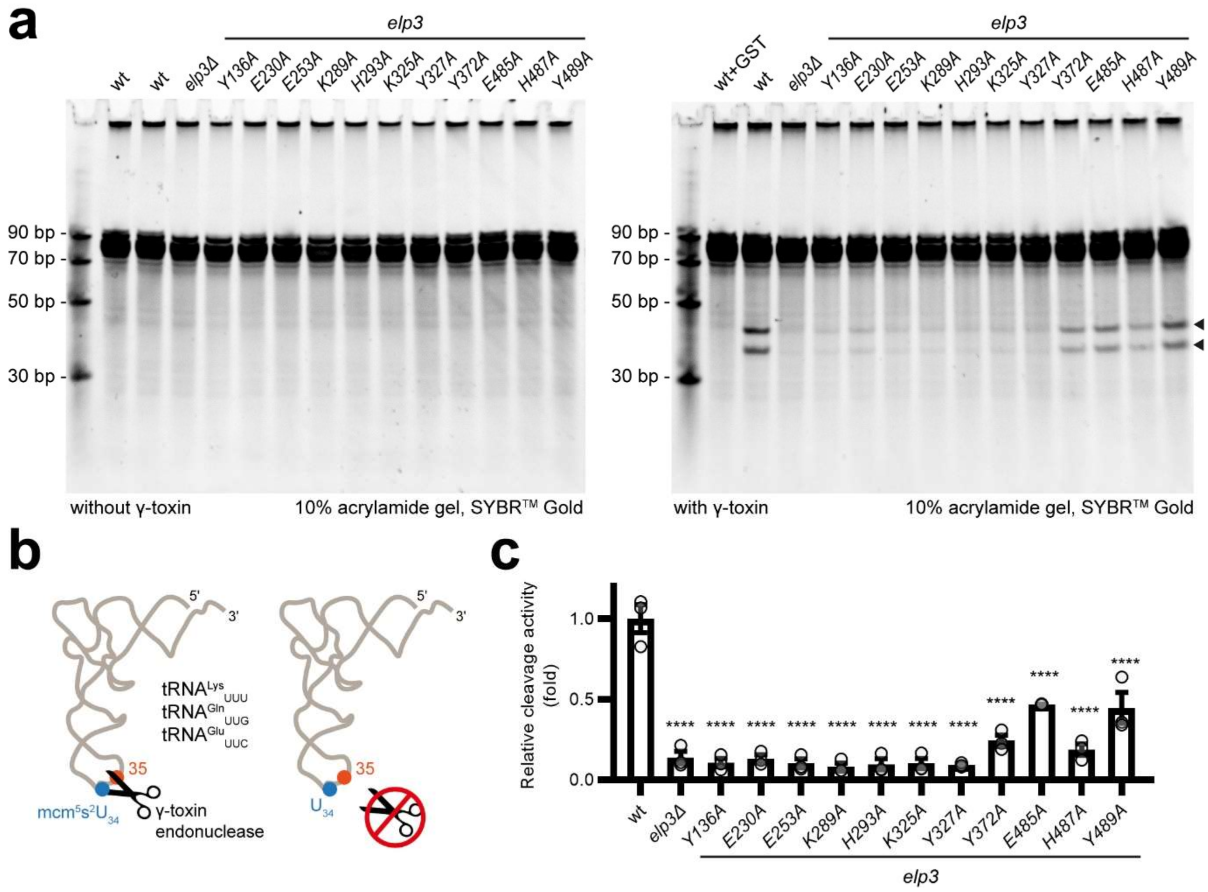

Fig S9. Elongator dependent anticodon cleavage assays with γ-toxin tRNase and bulk tRNAs isolated from yeast strains carrying *ELP3* gene mutations. (**a**) Representative TBE-UREA gels showing *in vitro* γ-toxin cleavage of isolated yeast bulk tRNA. The cleavage products are indicated by triangles. n = 3 (independent experiments).(**b**) tRNA recognition specificity for the γ-toxin tRNase. (**c**) Relative cleavage activity measured on tRNA from (**a**). n = 3 (independent experiments). Statistical analysis: one-way ANOVA. Statistically significant differences are indicated ($p \leq 0.0001$). Data are presented as mean values ± SEM. Source data are provided as a Source Data file.

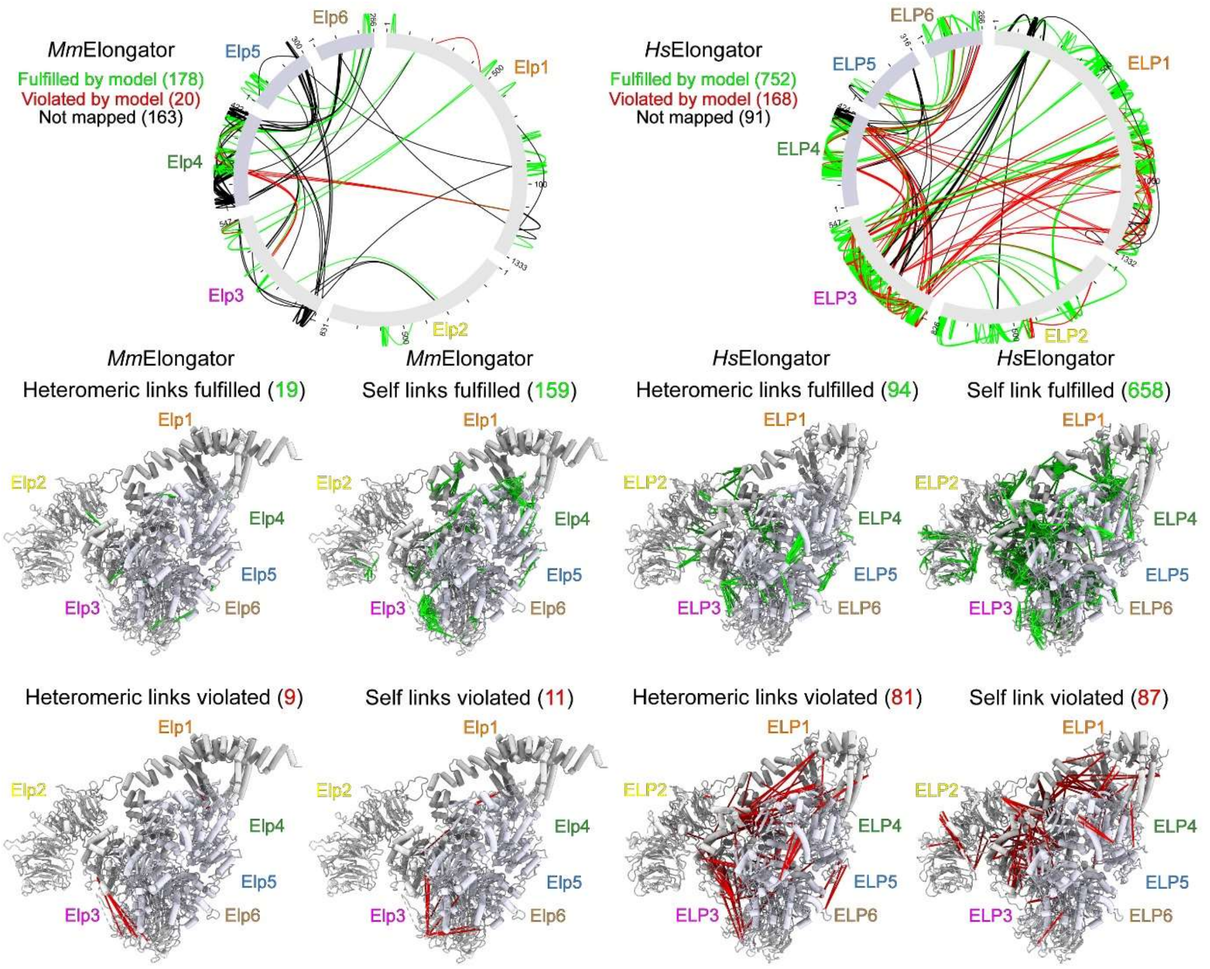

Fig S10. Crosslinking mass spectrometry analyses of the full assembled Elongator complex from mouse and human. Schematic representation of heteromeric- and self-links fulfilled by the model (green) or violated by the model (red) of mouse and human reconstituted Elongator. Links not mapped in the models are shown in black.

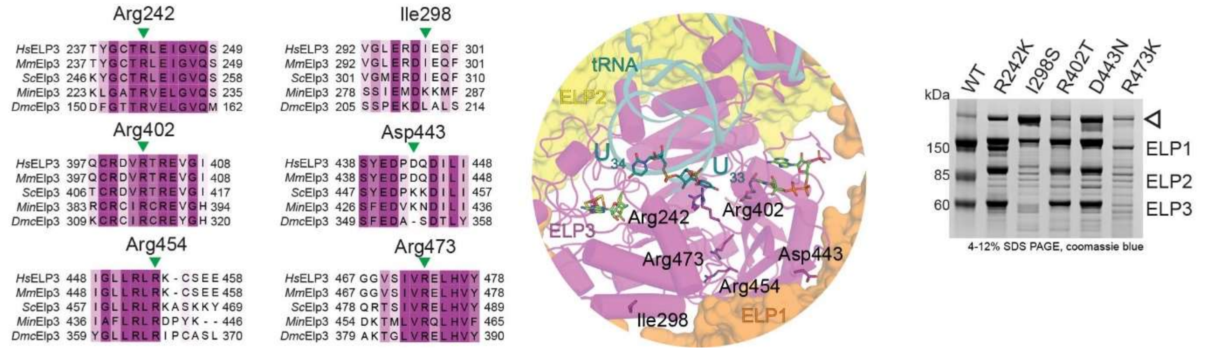

Fig S11. Analyses of clinically relevant mutations in ELP3. (left) Scheme of partial sequence alignments of the clinically relevant mutations of ELP3. (middle) Mapping of the clinically relevant mutations in ELP3 and the residues are highlighted. (right) SDS-PAGE gel analysis of the quality of wild-type (WT) and mutant ELP123 complexes. The non-specifically co-purified Acc1/ACACA protein[2] is indicated by the triangle. Source data are provided as a Source Data file.

Table S1. Yeast strains used and generated for *ELP3* gene mutagenesis and characterization.

Strain	Relevant genotype	Source
UMY2893	MATα *SUP4 leu2-3,112 trp1-1 can1-100 ura3-1 ade2-1 his3-11,15*	Ref [3]
ySF325	UMY2893, *ELP1-(c-myc)*$_{3x}$*::loxp, ELP5-FLAG*$_{3x}$*::loxp*	This study
ySF326	ySF325, *Δelp3::KlURA3*	This study
yPB48	ySF325, *elp3-Y136A::klTRP1*	This study
yPB49	ySF325, *elp3-E230A::klTRP1*	This study
yPB50	ySF325, *elp3-E253A::klTRP1*	This study
yPB51	ySF325, *elp3-K289A::klTRP1*	This study
yPB52	ySF325, *elp3-H293A::klTRP1*	This study
yPB53	ySF325, *elp3-K325A::klTRP1*	This study
yPB54	ySF325, *elp3-Y327A::klTRP1*	This study
yPB55	ySF325, *elp3-Y372A::klTRP1*	This study
yPB56	ySF325, *elp3-E485A::klTRP1*	This study
yPB57	ySF325, *elp3-H487A::klTRP1*	This study
yPB58	ySF325, *elp3-Y489A::klTRP1*	This study
yPB59	ySF325, *ELP3::klTRP1*	This study

Table S2. Strains used for *SUP4* purification.

Strain	Relevant genotype	Source
W303-1B	MATα *leu2-3,112 trp1-1 can1-100 ura3-1 ade2-1 his3-11,15*	Rodney Rothstein
ySF329	W303-1B, *Δelp3::KlURA3*	This study

Table S2. Plasmids used for *SUP4* purification, mutagenesis and cloning.

Plasmid	Properties	Source
YCplac111	*Amp^R CEN4 ARS1 ScLEU2*	Ref [4]
pSF305	*YCplac111, SUP4 +-200bp*	This study
pSF307	*pSF305, sup4-U33C*	This study
YEplac181	*Amp^R 2μ ScLEU2*	Ref [4]
pSF306	*Yeplac181, SUP4 +-200bp*	This study
pSF308	*pSF306, sup4-U33C*	This study

References

1. Pabis, M. *et al.* Molecular basis for the bifunctional Uba4–Urm1 sulfur-relay system in tRNA thiolation and ubiquitin-like conjugation. *EMBO J* **39**, e105087 (2020).
2. Jaciuk, M. *et al.* Cryo-EM structure of the fully assembled Elongator complex. *Nucleic Acids Res* **51**, 2011–2032 (2023).
3. Huang, B., Johansson, M. J. O. O. & Byström, A. S. An early step in wobble uridine tRNA modification requires the Elongator complex. *Rna* **11**, 424–436 (2005).
4. Gietz, R. D. & Akio, S. New yeast-Escherichia coli shuttle vectors constructed with in vitro mutagenized yeast genes lacking six-base pair restriction sites. *Gene* **74**, 527–534 (1988).
