# Supplementary material for: Cryo-EM structures of the human Elongator complex at work
Source: Nat Commun. 2024 May 15;15:4094. doi: 10.1038/s41467-024-48251-y (PMC11096365; doi:10.1038/s41467-024-48251-y)
Supplement: Supplementary file 3 — Description of Additional Supplementary Information [file 41467_2024_48251_MOESM3_ESM.pdf]

Description of Additional Supplementary Information

Supplementary Movie S1. Deformation of the ASL upon binding to ELP123, followed by the flipping out of U33.
