# Supplementary material for: Cryo-EM structures of the human Elongator complex at work
Source: Nat Commun. 2024 May 15;15:4094. doi: 10.1038/s41467-024-48251-y (PMC11096365; doi:10.1038/s41467-024-48251-y)
Supplement: Supplementary file 5 — Reporting Summary [file 41467_2024_48251_MOESM5_ESM.pdf]

nature portfolio

Corresponding author(s): Raffael Schaffrath, Ting-Yu Lin and Sebastian Glatt

Last updated by author(s): Apr 10, 2024

Reporting Summary

Nature Portfolio wishes to improve the reproducibility of the work that we publish. This form provides structure for consistency and transparency in reporting. For further information on Nature Portfolio policies, see our Editorial Policies and the Editorial Policy Checklist.

Statistics

For all statistical analyses, confirm that the following items are present in the figure legend, table legend, main text, or Methods section.

n/a	Confirmed	
☐	☒	The exact sample size (*n*) for each experimental group/condition, given as a discrete number and unit of measurement
☐	☒	A statement on whether measurements were taken from distinct samples or whether the same sample was measured repeatedly
☐	☒	The statistical test(s) used AND whether they are one- or two-sided<br>*Only common tests should be described solely by name; describe more complex techniques in the Methods section.*
☒	☐	A description of all covariates tested
☐	☒	A description of any assumptions or corrections, such as tests of normality and adjustment for multiple comparisons
☐	☒	A full description of the statistical parameters including central tendency (e.g. means) or other basic estimates (e.g. regression coefficient) AND variation (e.g. standard deviation) or associated estimates of uncertainty (e.g. confidence intervals)
☐	☒	For null hypothesis testing, the test statistic (e.g. *F*, *t*, *r*) with confidence intervals, effect sizes, degrees of freedom and *P* value noted<br>*Give P values as exact values whenever suitable.*
☒	☐	For Bayesian analysis, information on the choice of priors and Markov chain Monte Carlo settings
☒	☐	For hierarchical and complex designs, identification of the appropriate level for tests and full reporting of outcomes
☒	☐	Estimates of effect sizes (e.g. Cohen's *d*, Pearson's *r*), indicating how they were calculated

Our web collection on statistics for biologists contains articles on many of the points above.

Software and code

Policy information about availability of computer code

Data collection	Cryo-EM - EPU v. 2.10.0.1941REL (Thermo Fisher)<br>Biophysics - MO.Control v2.5.4 (NanoTemper Technologies GmbH), AN.Control Software v1.1 (NanoTemper TechnologiesGmbH)
Data analysis	cryo-EM - ChimeraX 1.2.5, CryoSPARC 3.3.0, DeepEMhancer 0.14, Durchlichtelektronenmikroskopiebilddatenentzerrungswerkzeug 1.0.9, Excel 365, ISOLDE 1.4, Namdinator 2.12, Phenix 1.19.2-4158, PyMOL 1.7, Relion 3.1, WinCOOT 0.9.7 EL<br>Biophysics - MO.Affinity Analysis v3.0.5, PR.PantaAnalysis v1.4.4 (NanoTemper Technologies), AN.StabilityAnalysis v1.1 (NanoTemper Technologies), GraphPad Prism (GraphPad Software), Excel365

For manuscripts utilizing custom algorithms or software that are central to the research but not yet described in published literature, software must be made available to editors and reviewers. We strongly encourage code deposition in a community repository (e.g. GitHub). See the Nature Portfolio guidelines for submitting code & software for further information.

Data

Policy information about availability of data

All manuscripts must include a data availability statement. This statement should provide the following information, where applicable:

- Accession codes, unique identifiers, or web links for publicly available datasets
- A description of any restrictions on data availability
- For clinical datasets or third party data, please ensure that the statement adheres to our policy

The micrographs, atomic coordinates and the cryo-EM map of the ELP123–tRNAGlnUUG–acetyl-CoA complex have been deposited at the Electron Microscopy Public Image Archive (EMPIAR-11650), the Protein Data Bank (PDB ID 8PTX) and the Electron Microscopy Data Bank (EMD-17924). The atomic coordinates and cryo-EM map of the ELP123, ELP123–tRNAGlnUUG–ECA and ELP123–tRNAGlnUUG–DCA have been deposited to the Protein Data Bank (PDB ID 8PTY, 8PTZ, 8PU0) and the Electron Microscopy Data Bank (EMD-17925, EMD-17926, EMD-17927), respectively. The crystal structure of tRNA (PDB 1EHZ) is available at https://www.rcsb.org/structure/1EHZ. The mass spectrometry data for ELP3 acetylation were deposited to the MassIVE repository with the dataset identifier MSV000092998 (doi:10.25345/C5N01044H). All crosslinking mass spectrometry data for mouse (ID JPST002342; https://repository.jpostdb.org/entry/JPST002342) and human (ID JPST002341; https://repository.jpostdb.org/entry/JPST002341) Elongator are available at (https://repository.jpostdb.org). Source data are provided with this paper.

Research involving human participants, their data, or biological material

Policy information about studies with human participants or human data. See also policy information about sex, gender (identity/presentation), and sexual orientation and race, ethnicity and racism.

Reporting on sex and gender	n/a
Reporting on race, ethnicity, or other socially relevant groupings	n/a
Population characteristics	n/a
Recruitment	n/a
Ethics oversight	n/a

Note that full information on the approval of the study protocol must also be provided in the manuscript.

Field-specific reporting

Please select the one below that is the best fit for your research. If you are not sure, read the appropriate sections before making your selection.

☒ Life sciences ☐ Behavioural & social sciences ☐ Ecological, evolutionary & environmental sciences

For a reference copy of the document with all sections, see nature.com/documents/nr-reporting-summary-flat.pdf

Life sciences study design

All studies must disclose on these points even when the disclosure is negative.

Sample size	For cryo-EM analyses, details of the sample size are listed in the Supplementary Figure S1-4 and was chosen so that high resolution structures are constructed.
Data exclusions	For cryo-EM analyses, several iterations of reference-free 2D class averaging and unsupervised 3D classification were used to remove suboptimal particles.
Replication	Biophysical measurements were repeated three times on different batches of in vitro transcribed tRNAs and purified protein complexes.
Randomization	No specific randomization was used in this study. Randomization is not relevant for this study because the experiments did not require allocation of individuals into groups.
Blinding	No specific blinding was used for the experiments presented in the study. Blinding is not relevant to cryo-EM and biophysical analyses in this study.

Reporting for specific materials, systems and methods

We require information from authors about some types of materials, experimental systems and methods used in many studies. Here, indicate whether each material, system or method listed is relevant to your study. If you are not sure if a list item applies to your research, read the appropriate section before selecting a response.

Materials & experimental systems

n/a	Involved in the study
☒	☐ Antibodies
☐	☒ Eukaryotic cell lines
☒	☐ Palaeontology and archaeology
☒	☐ Animals and other organisms
☒	☐ Clinical data
☒	☐ Dual use research of concern
☒	☐ Plants

Methods

n/a	Involved in the study
☒	☐ ChIP-seq
☒	☐ Flow cytometry
☒	☐ MRI-based neuroimaging

Eukaryotic cell lines

Policy information about cell lines and Sex and Gender in Research

Cell line source(s)	Yeast strain UMY2893 generated by Anders Bystrom (Huang et al. 2005). Sf9 insect cells and Hi5 cells purchased from ThermoFischer. Yeast strain W303-1B was constructed by Rodney Rothstein (Thomas & Rothstein, 1989; DOI: 10.1016/0092-8674(89)90584-9)
Authentication	MATα SUP4 leu2-3,112 trp1-1 can1-100 ura3-1 ade2-1 his3-11,15 and MATα leu2-3,112 trp1-1 can1-100 ura3-1 ade2-1 his3-11,15. None of the cell lines was authenticated, but all lines were purchased from commercial vendors, which documented and certified their identity upon delivery.
Mycoplasma contamination	Cell were not tested against mycoplasma contamination. No phenotypic experiments were carried out and cell lines were only used to produce/isolate purified proteins and tRNAs. No direct scientific conclusions were drawn from the behavior of the respective cell lines.
Commonly misidentified lines (See ICLAC register)	No commonly misidentified cell lines were used in this study.

Plants

Seed stocks	n/a
Novel plant genotypes	n/a
Authentication	n/a
